# Comparative effect of olmesartan and candesartan on lipid metabolism and renal function in patients with hypertension: a retrospective observational study

**DOI:** 10.1186/1475-2840-10-74

**Published:** 2011-08-10

**Authors:** Yayoi Nishida, Yasuo Takahashi, Tomohiro Nakayama, Masayoshi Soma, Satoshi Asai

**Affiliations:** 1 Division of Genomic Epidemiology and Clinical Trials, Advanced Medical Research Center, Nihon University School of Medicine, 30-1 Oyaguchi-Kamimachi, Itabashi-ku, Tokyo 173-8610, Japan; 2 Division of Clinical Trial Management, Advanced Medical Research Center, Nihon University School of Medicine, 30-1 Oyaguchi-Kamimachi, Itabashi-ku, Tokyo 173-8610, Japan; 3 Division of Pharmacology, Department of Biomedical Sciences, Nihon University School of Medicine, 30-1 Oyaguchi-Kamimachi, Itabashi-ku, Tokyo 173-8610, Japan; 4 Division of Laboratory Medicine, Department of Pathology and Microbiology, Nihon University School of Medicine, 30-1 Oyaguchi-Kamimachi, Itabashi-ku, Tokyo 173-8610, Japan; 5 Division of General Medicine, Department of Medicine, Nihon University School of Medicine, Tokyo, 30-1 Oyaguchi-Kamimachi, Itabashi-ku, Tokyo 173-8610, Japan

**Keywords:** angiotensin II receptor blocker (ARB), olmesartan, candesartan, lipid metabolism, renal function, retrospective observational study

## Abstract

**Background:**

Angiotensin II receptor blockers (ARBs), including olmesartan and candesartan, are widely used antihypertensive agents. Many clinical studies have demonstrated that ARBs have organ-protecting effects, e.g., cardioprotection, vasculoprotection and renoprotection. However, the effect of prolonged olmesartan monotherapy on lipid metabolism in patients with hypertension is less well studied. We performed a retrospective observational study to compare the effects of olmesartan with those of candesartan, focusing on lipid metabolism and renal function.

**Methods:**

We used data from the Clinical Data Warehouse of Nihon University School of Medicine obtained between Nov 1, 2004 and Feb 28, 2011, to identify cohorts of new olmesartan users (n = 168) and candesartan users (n = 266). We used propensity-score weighting to adjust for differences in all covariates (age, sex, comorbid diseases, previous drugs) between olmesartan and candesartan users, and compared serum chemical data including serum triglyceride (TG), LDL-cholesterol (LDL-C), total cholesterol (TC), potassium, creatinine and urea nitrogen. The mean exposure of olmesartan and candesartan users was 126.1 and 122.8 days, respectively.

**Results:**

After adjustment, there were no statistically significant differences in all covariates between olmesartan and candesartan users. The mean age was 60.7 and 61.0 years, and 33.4% and 33.7% of olmesartan and candesartan users were women, respectively. There were no statistically significant differences in mean values for all laboratory tests between baseline and during the exposure period in both olmesartan and candesartan users. In olmesartan users, the reduction of serum TG level was significant in comparison with that in candesartan users. Other parameters of lipid profile and renal function showed no statistically significant difference in the change from baseline to during the exposure period between olmesartan and candesartan users.

**Conclusions:**

In this study, we observed a more beneficial effect on lipid metabolism, a reduction of serum TG, with olmesartan monotherapy than with candesartan monotherapy. However, there were no clinically significant changes in the levels of all test parameters between baseline and during the exposure period with both drugs. These results suggest that the influence of olmesartan or candesartan monotherapy on lipid metabolism and renal function is small, and that they can be safely used in patients with hypertension.

## Introduction

Angiotensin II receptor blockers (ARBs) are widely used antihypertensive agents that act through inhibition of angiotensin II type 1 (AT_1_) receptors. In addition to antihypertensive effects, ARBs have been shown to have organ-protecting effects, including vasculoprotection [1], cardioprotection [2] and renoprotection [3,4]. Peroxisome proliferator activated receptor gamma (PPAR-γ), an intracellular receptor that regulates glucose and lipid metabolism, is modulated by different ARBs. Thereby, ARBs have been considered to improve insulin resistance [5,6], and we reported that monotherapy with ARBs had a favorable effect on glucose metabolism [7]. Differences in pharmacology and pleiotropic effects appear to exist across ARBs [5,6], which may be of clinical importance and help physicians make decisions on drug selection.

Hypertension and dyslipidemia are conditions that frequently coexist. They are both major determinants of cardiovascular disease, and together cause an increase in coronary heart disease-related events [8]. In experimental models, some ARBs have demonstrated the ability to affect lipid metabolism in a modest but significant way [9]. Whether ARBs have a favorable effect on lipid metabolism in humans may be of clinical significance, especially in treating patients with dyslipidemia. Candesartan, which binds more tightly to and dissociates more slowly from the AT_1 _receptor than other ARBs, had the weakest PPAR-γ modulatory activity [5,6]. Our recent study showed that candesartan monotherapy at a therapeutic dosage had a minimal effect on lipid metabolism for long periods up to 12 months [10]. Olmesartan medoxomil is an ARB that is characterized by strong blood pressure-lowering efficacy with a fast onset, prolonged duration of action and good tolerability. Olmesartan has moderate PPAR-γ modulator activity [5,6], and thereby has the possibility of affecting lipid metabolism. Some clinical studies have demonstrated that olmesartan has organ-protecting effects, including long-term renoprotection in patients with type 2 diabetes, and beneficial effects to reduce cardiovascular risk in patients with atherosclerosis [11,12]. A few clinical studies reported the effect of olmesartan on lipid metabolism [13-15]. However, their subjects were patients who were treated with olmesartan combined with other antihypertensive drugs or lipid-lowering drugs. Therefore, the effect of prolonged olmesartan monotherapy on lipid metabolism in patients with hypertension is less well studied. In this study, we examined changes in the plasma lipid profile of new users of generally prescribed doses of olmesartan medoxomil, and compared them with those in new users of candesartan cilexetil at doses that have previously shown a minimal effect on lipid metabolism. We also examined changes in serum potassium level in addition to serum creatinine level and serum urea nitrogen, as parameters of renal function.

## Methods

### Data source

This was a retrospective database study using the Nihon University School of Medicine (NUSM) Clinical Data Warehouse (CDW), which is a centralized data repository that integrates separate databases, such as an order entry database and a laboratory results database, from the hospital information systems at three hospitals affiliated with NUSM. The prescription database in the CDW contains information from over 0.5 million patients, and prescribing data are linked longitudinally to detailed clinical information such as patient demographics, diagnosis, and laboratory data. Several epidemiological studies examining the effects of antihypertensive drugs on metabolic and electrolyte changes using NUSM's CDW have been published [7,10,16].

### Study population

Patients with mild to moderate hypertension, aged over 20 years who had been newly treated with olmesartan medoxomil or candesartan cilexetil for at least four weeks between November, 2004 and February, 2011, were identified for the study. We identified 6,724 patients with olmesartan treatment and 11,069 patients with candesartan treatment who fulfilled the above criteria. We excluded patients who had been treated with other antihypertensive drugs (ARB other than olmesartan or candesartan, ARB combination drug, calcium channel blocker, angiotensin-converting enzyme inhibitor (ACEI), diuretic, alpha-blocker, beta-blocker, alpha and beta-blocker, alpha-agonist, reserpine, vasodilator, renin inhibitor) during the study period. We also excluded patients who had received the following drugs that affect the serum levels of parameters of lipid metabolism or renal function: lipid-lowering drugs, potassium preparations, and ion-exchange resins (e.g. sodium polystyrene sulfonate), in the 60 days preceding the date of a laboratory test. Consequently, the study cohorts included 168 new users of olmesartan monotherapy (who received 5-40 mg/day) and 266 new users of candesartan monotherapy (who received 1-12 mg/day). The experimental protocol was approved by the Ethical Committee of Nihon University School of Medicine.

### Exposure and measurements

The baseline measurement period (non-exposure period) was defined as 90 days before the start of olmesartan or candesartan monotherapy. The exposure period (outcome measurement period) was defined as between 1 and 6 months after the start of olmesartan or candesartan monotherapy. The mean exposure of olmesartan users and candesartan users was 126.1 days and 122.8 days, respectively. Blood test data, including serum low-density lipoprotein-cholesterol (LDL-C), triglyceride (TG), total cholesterol (TC), potassium, creatinine and urea nitrogen, were collected for each individual at the date nearest the start of olmesartan or candesartan monotherapy in the baseline period, and at the date nearest six months after the start of olmesartan or candesartan monotherapy in the exposure period.

### Data elements

For each patient, we collected information of patient demographics (age and sex), medical history, use of drugs, and laboratory results. Medical history included cerebrovascular disease (ICD-10 code, I60-I69), ischemic heart disease (I20-I25), other heart disease (I30-I52), liver disease (K70-K77), kidney disease (N00-N19), gout (M10), thyroid gland disorder (E00-E07), hyperlipidemia (E78.0-E78.5), diabetes mellitus (E10-E14), and malignant neoplasm (C00-C97) diagnosed in the 365 days preceding the first date of prescription of olmesartan or candesartan. Drugs used during the 60 days before the start of olmesartan or candesartan monotherapy included chemotherapeutic drugs, immunosuppressive drugs, steroids, insulin, oral hypoglycemic drugs, and thyroid drugs.

### Statistical analysis

All statistical analyses were performed with SAS software, version 9.1.3 (SAS Institute Inc., Cary, NC). To compare differences in baseline characteristics, we used *t*-test for continuous variables and chi-squared test for categorical data. Adjustments for differences as observed in Table 1 were performed using a propensity-score weighting technique to balance treatment groups and address the potential for treatment selection bias [17-19]. This method is also known as the inverse probability of treatment weighted (IPTW) estimator, described by Robins and colleagues [20]. To apply this method, the propensity score for each subject was obtained by fitting a logistic regression model that includes the predictor variable (i.e., olmesartan user or candesartan user) as an outcome and all baseline covariates as shown in Table 1. After the propensity score was constructed, we calculated the propensity score weight as the inverse of the propensity score. Paired *t*-test and a propensity score-weighted *t*-test were used to compare the differences in means between baseline and during the exposure period, and between olmesartan users and candesartan users, respectively. All reported p-values are two sided. A result was considered statistically significant if the p value was less than 0.05.

**Table 1:** Baseline characteristics of patients before and after IPTW adjustment

	Number of patients		Percent distribution*			
		
Characteristics	Olmesartan (n = 168)	Candesartan (n = 266)	Before adjustment		After adjustment	
			Olmesartan	Candesartan	Olmesartan	Candesartan
Age (years, mean ± SD)			61.6 ± 12.4	60.9 ± 3.7	60.7 ± 13.9	61.0 ± 13.4
Sex (women, %)	55	96	32.7	36.1	33.4	33.7
Medical history						
Cerebrovascular disease	30	49	17.9	18.4	18.5	17.7
Ischemic heart disease	39	34	23.2†	12.8	17.5	16.7
Other heart disease	55	87	32.7	32.7	33.2	32.6
Liver disease	61	99	36.3	37.2	37.4	36.4
Kidney disease	76	104	45.2	39.1	43.9	42.1
Thyroid disease	29	61	17.3†	22.9	21.7	20.6
Diabetes mellitus	141	171	83.9†	64.3	72.6	71.8
Hyperlipidemia	144	215	85.7	80.8	82.2	82.6
Gout	2	12	1.2†	4.5	1.6	3.2
Malignant neoplasm	49	99	29.2†	37.2	36.0	34.7
Previous drugs						
Chemotherapeutic drugs	2	3	1.2	1.1	0.9	1.0
Immunosuppressive drugs	2	2	1.2	0.8	0.8	0.8
Steroids	8	14	4.8	5.3	5.1	5.1
Thyroid drugs	5	8	3.0	3.0	3.2	3.0
Insulin	26	21	15.5†	7.9	10.4	10.1
Oral hypoglycemic drugs	54	64	32.1†	24.1	27.4	27.5

*Data are percent distribution of patients unless otherwise stated. †p < 0.05 (candesartan vs olmesartan).

## Results

Table 1 shows the characteristics of the patients who had been treated with olmesartan monotherapy or candesartan monotherapy, before and after IPTW adjustment. Before adjustment, olmesartan users were more likely to have ischemic heart disease and diabetes, utilizing more antidiabetic agents including insulin and oral hyperglycemic drugs, than candesartan users. After IPTW adjustment, the mean age was 60.7 and 61.0 years, and 33.4% and 33.7% of olmesartan and candesartan users were women, respectively. There was no statistically significant difference in all covariates between olmesartan users and candesartan users.

Table 2 shows the results of laboratory tests at baseline and during the exposure period. There were no statistically significant differences in the mean values for all laboratory tests between baseline and during the exposure period in both olmesartan users and candesartan users. Serum urea nitrogen and creatinine levels tended to increase in both users. Serum TG level tended to decrease in olmesartan users, while it tended to increase in candesartan users. Mean values of all laboratory tests remained within normal limits during the study period in both olmesartan users and candesartan users.

**Table 2:** Summary of serum chemical data

Laboratory test	Olmesartan (n = 168)				Candesartan (n = 266)			
		
	Mean	95% CI		p-value	Mean	95% CI		p-value
						
		Lower	Upper			Lower	Upper	
Lipid metabolism								
Triglyceride (mmol/L)								
Baseline	1.87	1.68	2.06		1.69	1.48	1.91	
Exposure	1.76	1.58	1.94	0.3912	1.81	1.65	1.97	0.3862

LDL-cholesterol (mmol/L)								
Baseline	3.01	2.88	3.14		3.14	3.05	3.24	
Exposure	2.93	2.81	3.05	0.3609	3.09	2.99	3.19	0.4478

Total cholesterol (mmol/L)								
Baseline	5.25	5.10	5.40		5.34	5.21	5.46	
Exposure	5.14	4.99	5.28	0.2871	5.31	5.19	5.43	0.7509

Kidney function								
Urea nitrogen (mmol/L)								
Baseline	5.63	5.29	5.97		5.75	5.49	6.00	
Exposure	5.92	5.50	6.34	0.2969	6.03	5.71	6.34	0.1743

Creatinine (μmol/L)								
Baseline	70.00	66.57	73.44		75.58	72.04	79.13	
Exposure	72.31	68.66	75.95	0.3645	78.20	74.11	82.30	0.3416

Potassium (mmol/L)								
Baseline	4.40	4.34	4.47		4.32	4.27	4.36	
Exposure	4.47	4.41	4.54	0.1161	4.36	4.31	4.41	0.2339

p value: baseline vs exposure. CI denotes confidence interval. The mean values of all parameters showed no significant change between baseline and during the exposure period.

Table 3 shows the mean changes in the values of laboratory test parameters during the exposure period compared with baseline after IPTW adjustment. In olmesartan users, the reduction of serum TG level was significant in comparison with that in candesartan users. Other tests showed no statistically significant difference in the change from baseline to during the exposure period between olmesartan users and candesartan users. We further examined the differences between olmesartan users and candesartan users in patients with a previous diagnosis of diabetes or hyperlipidemia (Table 4), because these diseases are leading risk factors for cardiovascular disease. We found the same outcome in patients with diabetes or hyperlipidemia as that in the overall population, with a significant reduction of serum TG level in olmesartan users in comparison with candesartan users.

**Table 3:** Changes in adjusted laboratory test values during exposure period from baseline

Laboratory tests	Olmesartan (n = 168)			Candesartan (n = 266)			p value
			
	Mean	95% CI		Mean	95% CI		
					
		Lower	Upper		Lower	Upper	
Lipid metabolism							
Δ Triglyceride (mmol/L)	-0.080	-0.185	0.024	0.132	0.025	0.239	0.0087*
Δ LDL-cholesterol (mmol/L)	-0.085	-0.146	-0.023	-0.065	-0.112	-0.017	0.6094
Δ Total cholesterol (mmol/L)	-0.091	-0.168	-0.015	-0.039	-0.106	0.028	0.3248
Renal function							
Δ Urea nitrogen (mmol/L)	0.381	0.181	0.582	0.242	0.072	0.412	0.3059
Δ Creatinine (μmol/L)	2.378	1.264	3.492	2.608	1.397	3.828	0.7989
Δ Potassium (mmol/L)	0.078	0.035	0.122	0.042	0.009	0.076	0.1933

Δ indicates the mean change in laboratory test value during the exposure period from baseline. CI denotes confidence interval. *: p < 0.05 (candesartan vs olmesartan)

**Table 4:** Changes in adjusted laboratory test values during exposure period from baseline in patients with diabetes mellitus or hyperlipidemia

Category	Laboratory tests	Olmesartan			Candesartan			
				
		Mean	95% CI		Mean	95% CI		p value
						
			Lower	Upper		Lower	Upper	
Diabetes mellitus	Lipid metabolism							
	Δ Triglyceride (mmol/L)	-0.096	-0.194	0.003	0.108	-0.022	0.237	0.0228*
	Δ LDL-cholesterol (mmol/L)	-0.117	-0.183	-0.051	-0.101	-0.160	-0.041	0.7241
	Δ Total cholesterol (mmol/L)	-0.163	-0.246	-0.080	-0.089	-0.172	-0.005	0.2313
	Renal function							
	Δ Urea nitrogen (mmol/L)	0.220	0.018	0.422	0.251	0.019	0.483	0.8514
	Δ Creatinine (μmol/L)	2.298	1.132	3.456	3.209	1.618	4.791	0.4025
	Δ Potassium (mmol/L)	0.047	-0.001	0.096	0.060	0.017	0.104	0.7

Hyperlipidemia	Lipid metabolism							
	Δ Triglyceride (mmol/L)	-0.079	-0.203	0.044	0.106	-0.019	0.231	0.0498*
	Δ LDL-cholesterol (mmol/L)	-0.108	-0.177	-0.038	-0.087	-0.139	-0.036	0.6414
	Δ Total cholesterol (mmol/L)	-0.111	-0.196	-0.026	-0.060	-0.135	0.014	0.3877
	Renal function							
	Δ Urea nitrogen (mmol/L)	0.401	0.174	0.629	0.231	0.034	0.428	0.2755
	Δ Creatinine (μmol/L)	2.157	0.857	3.448	2.661	1.308	4.005	0.6173
	Δ Potassium (mmol/L)	0.029	-0.015	0.073	0.049	0.012	0.086	0.5099

Δ indicates the mean change in laboratory test value during the exposure period from baseline. CI denotes confidence interval. *: p < 0.05 (candesartan vs olmesartan)

## Discussion

In this study, we evaluated and compared the effects of olmesartan and candesartan monotherapy on lipid metabolism and renal function. We found that the reduction of serum TG level in olmesartan users was significantly greater than that in candesartan users, although there were no significant differences in the mean values of all test results between baseline and during the exposure period in both users. These results suggest minimal effects of olmesartan monotherapy on lipid metabolism and renal function, the same as with candesartan monotherapy. The results also suggest that olmesartan monotherapy may have a more beneficial effect on TG metabolism than candesartan monotherapy. Stratified analysis showed the same outcome in patients with diabetes or hyperlipidemia as that in the overall population, with a significant reduction of serum TG level in olmesartan users in comparison with candesartan users. These results suggest a more beneficial effect of olmesartan monotherapy on TG metabolism than candesartan monotherapy in patients with diabetes or hyperlipidemia.

The lipid-lowering property of ARB is possibly due to numerous different mechanisms. It is well known that some ARBs modulate PPAR-γ, which regulates lipid metabolism [5,6]. It is more likely that some ARBs such as telmisartan can activate PPAR-γ, which partially reduces TG and LDL-C levels. Additionally, an experimental study showed that dyslipidemia may activate angiotensin II endothelial injury and lipid peroxidation by an AT_1 _receptor-mediated mechanism, suggesting an interaction between the renin-angiotensin-aldosterone system and lipid metabolism [21]. Some clinical studies have shown a close relationship between AT_1 _receptor density and plasma LDL-C, and an association of the use of statins to lower cholesterol with AT_1 _receptor down-regulation [22,23]. It is possible that differences in PPAR-γ activation or AT_1 _blockage cause the differences in the lipid-lowering effect across different ARBs, partially resulting in the significant difference in reduction of serum TG level between olmesartan users and candesartan users in our study. PPAR-γ activation occurs at therapeutic dosages only with telmisartan, which is the strongest modulator of PPAR-γ, but cannot be achieved with other ARBs at therapeutic dosages [5]. Therefore, the effects of olmesartan and candesartan on lipid metabolism via PPAR-γ activation may be very small at therapeutic dosages. Supporting this, our study showed no significant change of serum LDL-C, TC, and TG levels from baseline to the end of the exposure period in both olmesartan and candesartan users. Also, several clinical studies showed similar results with administration of candesartan, suggesting no effect on lipid metabolism in patients with mild hypertension or/and type 2 diabetes [24-27]. On the other hand, only a few studies have examined the effect of olmesartan on lipid metabolism. Fliser et al showed no significant changes of HDL-C, LDL-C, TC and TG levels after 6 weeks of olmesartan administration to patients with essential hypertension and microinflammation [13]. Our results, in combination with a previous study, suggest a lack of obvious evidence of an unfavorable influence of olmesartan on lipid metabolism. Therefore, the influence of olmesartan monotherapy on lipid metabolism may be small and may not be of clinical concern, the same as with candesartan monotherapy.

Clinical trials have shown that ARBs have renoprotective effects in diabetes and can slow the progression of microalbuminuria [3,28,29]. Although ARBs have a significant antiproteinuric effect, there may be concern about complications, including increased serum creatinine level and hyperkalemia. In the CHARM (Candesartan in Heart failure-Assessment of Reduction in Mortality and Morbidity) study, the risk of hyperkalemia increased with addition of candesartan [30]. In the CHARM-Added trial and CHARM-Overall trial, more patients discontinued candesartan than placebo because of adverse effects, particularly hypotension, increased serum creatinine and hyperkalemia [2,31]. In this study, serum urea nitrogen and creatinine levels tended to increase from baseline, but were not significant in both olmesartan and candesartan users. Also, we showed no significant change of potassium level in both olmesartan and candesartan users. Our study, showing no clinically significant aggravation of renal function, suggests that olmesartan or candesartan monotherapy may be safely used for patients with hypertension. The reason for this discrepancy between our study and those trials may be variations in study design and cofounding variables. In the CHARM-Added trial and CHARM-Overall trial, the candesartan group included patients with ACEI treatment. Although combination therapy with an ARB and an ACEI has greater improvement with regard to target organ damage, especially heart failure and proteinuria [32], it may be associated with complications including worsening of renal function and increased serum potassium level. Therefore, when prescribing an ARB, regular checks of serum potassium and creatinine levels should be performed, especially when combined with an ACEI.

Our study has some limitations. First, the retrospective and non-randomized nature of the design involved inherent issues of selection bias and confounding. We used rigorous statistical methods to balance potential confounding variables between olmesartan and candesartan users, including IPTW adjustment. However, their ability to control for differences was limited to variables that were available or measurable. Furthermore, the possibility that the findings of comparison of baseline and exposure period in each treatment group may be confounded by other variables should be considered when interpreting the results. Second, we did not fix the daily dosage in both olmesartan and candesartan users, because the achievement of blood pressure goal requires various doses of an agent across different individuals or even in the same individual in clinical practice. This study was not designed to assess the effects of olmesartan and candesartan at each dosage, because it is difficult to determine whether or not pharmacodynamics are dose-dependent in clinical settings. However, the findings of our study, using a sophisticated statistical method in a real-world setting, are reliable and informative for clinicians.

## Conclusion

In this study, we observed a more beneficial effect on lipid metabolism, a reduction of serum TG, with olmesartan monotherapy than with candesartan monotherapy. In addition, we observed no clinically significant changes of serum TG, TC, LDL-C, urea nitrogen, creatinine, and potassium levels during olmesartan or candesartan treatment from baseline. These results suggest that the influence of olmesartan or candesartan monotherapy on lipid metabolism and renal function is small, and that they can be safely used for patients with hypertension.

## Competing interests

The authors declare that they have no competing interests.

## Authors' contributions

YN and YT conceived the study and participated in its design. YN performed the statistical analyses. YN and YT drafted the manuscript. TN, MS and SA interpreted the data. All authors have read and approved the final manuscript.
